# From Genes to Ecosystems: Marc Van Montagu on Science, Society, and the Future of Life

**DOI:** 10.1002/ggn2.70047

**Published:** 2026-09-26

**Authors:** Marc Van Montagu

**Affiliations:** ^1^ Department of Plant Biotechnology and Bioinformatics Ghent University Ghent Belgium; ^2^ Center for Plant Systems Biology VIB Ghent Belgium

## Abstract

Editor's Note: As part of the Advanced Dialogues series, the editor invites leading scientists and emerging voices to share their research journeys, experiences, and visions for the future of their fields. In this feature, Marc Van Montagu—Emeritus Professor at Ghent University, a founding father of plant genetic engineering, and Chairman of IPBO‐VIB, shares his insights at 92, drawing on a personal conversation with the editor and a lecture he delivered at the ICG‐20 conference, “From Feral to Fertile: Awakening Life in the Desert”, in Hangzhou.

## Abstract 2

Marc Van Montagu, Emeritus Professor at Ghent University, a founding father of plant genetic engineering, shares his insights at 92, drawing on a personal conversation with the editor and a lecture he delivered at the ICG‐20 conference, “From Feral to Fertile: Awakening Life in the Desert,” in Hangzhou.

## Fifty Years of Transgenic Plants — and What We Have Not Yet Understood

When we talk about genetics, we must think back to the very beginning — the understanding of what we should not do. When we think of Darwin, he drew our attention to evolution. Understanding evolution is fundamental before we can truly progress in genetics; we still have a lot of basic work to do. I refer here to François Jacob's book on the possibilities that nature has for developing genetics, a reminder that the best science begins not with a technique but with a question about what nature is actually doing [1].

Plants emerged from evolution and do move. They adapt to the signals around them, survive, and have created many things essential for human society. And as soon as the first genetic engineering became possible and a way was found to introduce new genes into plants [2, 3], society said: “You are doing dangerous things.” Industry said: “We have a specialty, and we don't want anybody fiddling around with genes.” The results were real nonetheless — a plant expressing a toxin from *Bacillus thuringiensis* provided insect resistance [4], and the barnase‐barstar chimaeric gene system enabled controlled male sterility in canola, transforming hybrid seed production [5, 6]. At the moment, 173 transgenic crop events are approved in the EU [7], almost all for import as food or feed; one is authorised for cultivation. We see the problems of science being understood by society, and how we can move forward.

“*The reason why we urgently need to change our attitude is to understand what nature truly is — and how plants interact with the soil*.”

## The Forgotten Frontier: Soil as a Living Interface

Soil complexity was the twentieth century's blind spot. We focused on the plant and treated the ground beneath it as a substrate to fertilize and keep moist. We missed the fact that the soil is itself a living system, with a structure and chemistry as intricate as anything inside the plant. If you start studying the soil, you see very strange things. If you have sand and want to make the desert green again, you have problems. You see how the particulate matter diminishes and how the organic matter is further down. So you have to organize your thinking: what can we do with the soil if we want to make this barren soil fertile again?

People have studied clay and found that it has nanotubes. Take Montmorillonite, a soil clay mineral that retains water, whose layered structure creates nanoscale interplayer spaces where chemical reactions proceed very differently from those in free solution, in ways that mirror the environment inside an enzyme's active site. It is observed that protein molecules have a certain space called the active center, a kind of nanotube. A whole new organic chemistry has developed which we have been using for quite some time without fully realizing it. Discoveries in reticular chemistry and Metal‐Organic Frameworks reveal a continuum between organic and inorganic matter: soil's nanoconfined spaces act as powerful catalysts, facilitating reactions that mirror enzymatic functions in living cells. Omar M. Yaghi, Susumu Kitagawa and Richard Robson, were jointly awarded the Nobel Prize in Chemistry 2025 for the development of metal‐organic frameworks; from these porous materials, you can really make new kinds of compounds [8, 9]. The line between geology and biology is not where we thought it was.

The Egyptians already knew something of this. They used to take the silt along the River Nile and mix it with clay, because clay is truly the material where organic matter grows. A Norwegian company, Desert Control, has now made that industrial: their liquid nanoclay penetrates sandy ground to root depth, typically 30 to 60 cm, binding sand particles through Van der Waals forces and creating conditions where organic matter can accumulate. Traditional farmers in Africa have long made charcoal and brought it into the soil. But it is industry that has to take over at scale. Can we do it better than the ancient Egyptians did?

## Do not Plant a Forest — Build a Community

We know that plant‐microbe interactions truly shape our planet, our agriculture, and our use of plants. Plants did not colonize land alone — the plant‐microbe holobiont functions as a single adaptive unit: bacteria and fungi that fix nitrogen, solubilize phosphate, create biofilm and hyphal networks around the roots, and exchange signaling molecules that coordinate the plant's response to stress. When we talk about restoring degraded land, we cannot talk only about the plant. We have to talk about the community the plant brings with it, and the community the soil needs to offer in return.

The data show that we simply cannot plant a new forest in pure sand; it will never work. You have to understand the function. Pioneer grasses, Stipagrostis species, can establish in near‐pure sand; they modify the soil for facilitative shrubs, which modify it for drought‐tolerant trees like Acacia, and so on. Use these plants to bring the first biomaterial, and then, by better understanding their functions, see what fundamental science can bring for the restoration of areas that formerly had plants. Don't plant a forest — build a community. Use the function, but choose the local form.

Biotechnology does not replace that local form, it amplifies it. The native plant is the base genome: already rooted in the local environment, adapted to local stress. Precision optimizations — improved drought resilience, stress‐resilient metabolism, engineered symbiosis, can be introduced without replacing the plant's ecological identity. The engineered microbes are the symbiotic partners: designed for nitrogen fixation, phosphate solubilization, drought resistance, and root signaling. The Integrated Restoration Engine works in four layers: Physical Foundation — clay minerals, biochar, inorganic soil amendments — then the Biotechnology Accelerator, then the Biological Catalyst, and at the top, Guiding Intelligence: precision genomics and AI, which optimize every decision below. Biotechnology doesn't replace nature. It amplifies its intelligence.

*“So I think it's time we stop cutting down the tropical forest and see, with the areas we have already destroyed, what science can bring us. And again, it will be genetics and AI that enable good reforestation*.”

## How Science Is Actually Done

If you ask me about the turning points in my scientific life, the first thing that comes to mind is learning to interact with people. I was an only child. My parents both worked, so I spent much of my time alone. It took time before I developed the habit of reaching out. I always learned best by listening. When people tell a story, I remember it.

Once you are in a lab, you have to learn to be precise, and that is not easy when everything is scarce. There were no commercial pipettes; they were glass, and you had to calibrate each one yourself. So, we all learned glass blowing and made our own flasks. There were no isotopes available in Belgium; when they came, you had to work with small amounts and be careful. And that was a pleasure, like a magician who does tricks, something mysterious. That brought genuine pleasure.

One of the earliest techniques I used to characterize an enzymatic reaction was manometry: a U‐tube with enzyme solution in one arm and substrate in the other, sealed inside a large aquarium set up in a deep old cellar with double doors so there was no draught; otherwise you would have false results. You waited for the temperature to stabilize, then slowly mixed the two solutions and watched a capillary tube to see whether the volume was growing or not. It is the same measurement you now do in a kit with immediate results — only it took many, many hours, also in the evening. If you were in the lab, you tried to be early and stay late. That was a habit.

What drives discovery? Curiosity. You see curious people around you, and you become curious yourself. Finding an answer is a pleasure. You watch others struggle with a problem and then ask the next question: Why is it like that? How does it happen? Science is a little like art. People search for something they do not yet fully understand, and gradually they find meaning in it. Sometimes others are impressed by your conclusions; sometimes they dismiss them or tell you that you have made a mistake. That is how you grow up as a scientist. You learn from those who have been there longer, who know more. There is a lot of imitation, but also a lot of invention.

The laboratory in Brussels was much more advanced in molecular biology at that time, and when a seminar speaker came from the United States, it was an event. Four or five of us would squeeze into a car and drive there just to listen. You watched someone reason carefully through a problem and learned how scientific thinking worked. Then one day you contributed an idea of your own, and others were surprised. That is when you learn what discussion really means.

A large part of the laboratory was devoted to journals, with complete collections stretching back to the nineteenth century. If you wanted a copy of a paper, you photographed it, developed the film in a darkroom, and printed it yourself. Later, those libraries disappeared because everything became electronic. Each approach has its costs.

## How Science Has Changed — and What Has Not

When I started in the lab, telephoning another country was exceptional; you had to write each call in the ledger because it was a billable expense. Nobody had money; there was not much money for research after the war. Everything had to be cheap. You could not simply buy and open a kit; they didn't exist. Each balance had to be calibrated; each solution made correctly. In the 1960s, sequencing a short RNA genome required glass columns two or three meters high, with a carousel of collection tubes, everything separated manually, fraction by fraction. People in the 1970s asked whether sequencing machines would ever work. Now we have the sequences of all the macromolecules. Big machines do in an afternoon what took a team a year.

What worries me is that there are now so many more recipes. You can do PCR without knowing why. The technique has outrun the thinking. When you had to make every solution by hand, you had to understand what you were doing. The question is whether that understanding is being maintained as the tools become more powerful and the kits more convenient.

What has not changed, and must not change, is the question itself. The same curiosity that drove us in a laboratory with no money, where everything had to be built by hand, needs to drive the work now. The tools have changed beyond recognition. The question has not.

## What I Would Say to Young Researchers

Trust in rationality and science; that is what brings innovation. But do not forget the society where you live. Happiness comes through the good application of rationality, and through understanding what life is and what the human place is — not only chemistry and physics, but all the human sciences. You have to do the thinking, but you must constantly assess: where is this thinking leading? Am I disturbing my community, the rest of the planet, other humans?

Be aware that aesthetics, the pleasure of the different arts, is also a form of rationality related to how you behave. Beware of prejudice; you see how prejudice gives errors in reasoning about materials and compounds; it does exactly the same when you are thinking about your own life, what you call happiness, empathy, and getting along with others.

You can only realize this by communicating with others; you have to convince others with facts; that is the best way. Things change: it is not a mechanical system; it is a dynamic situation. Once you understand something, you apply it, you react, and so you are changing something you cannot predict. You have to learn to adapt. Learning is not just describing what is, because meanwhile it is already different.

People often ask me about social media. It can be useful, especially for those who might otherwise struggle to express themselves. But it can also create many superficial connections. Real understanding still comes from investing time in people, listening to them, and learning from them.

Nothing is in the category of absolute black or white. If you think it is, you fool yourself. Act for positive things and not out of pessimism or aggression. Appreciate the diversity. The way you do the interaction is what defines it.

*“You have to think — but watch what you think, because you can run into trouble. But if you don't think, you will surely be in trouble. So that is why: do science, and see what questions can bring answers that are pleasing and help us. Be optimistic; otherwise, you are on the wrong track.”*

This article was edited by Yuming Hu, Wiley.

## Conflicts of Interest

The author declares no conflict of interest.

## References

[1] F. Jacob , Le Jeu des possibles: Essai sur la diversité du vivant (Fayard, 1981), English translation: *The Possible and the Actual* (Pantheon Books, 1982).

[2] L. Herrera‐Estrella , A. Depicker , M. Van Montagu , and J. Schell , “Expression of Chimaeric Genes Transferred Into Plant Cells Using a Ti‐Plasmid‐Derived Vector,” Nature 303 (1983): 209–213, 10.1038/303209a0. 1422044

[3] P. Zambryski , H. Joos , C. Genetello , J. Leemans , M. Van Montagu , and J. Schell , “Ti Plasmid Vector for the Introduction of DNA Into Plant Cells Without Alteration of Their Normal Regeneration Capacity,” EMBO Journal 2 (1983): 2143–2150, 10.1002/j.1460-2075.1983.tb01715.x. PMC555426 16453482

[4] M. Vaeck , A. Reynaerts , H. Höfte , et al., “Transgenic Plants Protected From Insect Attack,” Nature 328 (1987): 33–37, 10.1038/328033a0.

[5] C. Mariani , M. De Beuckeleer , J. Truettner , J. Leemans , and R. B. Goldberg , “Induction of Male Sterility in Plants by a Chimaeric Ribonuclease Gene,” Nature 347 (1990): 737–741, 10.1038/347737a0.

[6] C. Mariani , V. Gossele , M. De Beuckeleer , et al., “A Chimaeric Ribonuclease‐Inhibitor Gene Restores Fertility to Male Sterile Plants,” Nature 357 (1992): 384–387, 10.1038/357384a0.

[7] ISAAA GM Approval Database, https://www.isaaa.org/gmapprovaldatabase/.

[8] The Nobel Prize in Chemistry 2025 , S. Kitagawa , R. Robson , and O. M. Yaghi , For the Development of Metal‐Organic Frameworks (The Royal Swedish Academy of Sciences, 2025), https://www.nobelprize.org/prizes/chemistry/2025.

[9] H. Kim , S. Yang , S. R. Rao , et al., “Water Harvesting From Air With Metal‐Organic Frameworks Powered by Natural Sunlight,” Science 356, no. 6336 (2017): 430–434, 10.1126/science.aam8743. 28408720

